# Performance and Safety of Smooth-Collar (Tissue-Level) Implants: A Case Report and Clinical Literature Review

**DOI:** 10.7759/cureus.108268

**Published:** 2026-05-04

**Authors:** Fábio Guerra, Umberto Ramos, Irlan A Freires, Bruna Ghiraldini, Roberto S Pessoa, Guilherme Oliveira

**Affiliations:** 1 Implantology, Private Clinic, São José do Rio Preto, BRA; 2 Dental Hygiene, University of Doha for Science and Technology, Doha, QAT; 3 Clinical Dentistry, School of Dentistry, Universidade Federal do Espirito Santo, Vitória, BRA; 4 Dentistry, University of Florida College of Medicine, Jacksonville, USA; 5 Periodontology, Universidade Paulista (UNIP), São Paulo, BRA; 6 Periodontology, Centro Universitário do Triângulo (UNITRI), Uberlândia, BRA; 7 Periodontics, Universidade Federal de Uberlândia (UFU), Uberlândia, BRA

**Keywords:** dental implants, marginal bone loss, peri-implantitis, pterygoid implants, smooth collar, systematic review, tissue-level implants, zygomatic implants

## Abstract

The aim of the study is to describe a case series of full-arch rehabilitation of atrophic maxillae using smooth/machined collar (tissue-level) implants, including zygomatic and pterygoid implants, without bone grafting. Four consecutive patients with severe maxillary atrophy underwent clinical and radiographic evaluation, including cone-beam computed tomography, followed by implant placement using standardized surgical protocols. Immediate loading was performed when insertion torque exceeded 45-50 Ncm. Case 1 (65-year-old woman) was treated with four zygomatic, one pterygoid, and one conventional implant. Case 2 (58-year-old woman with controlled systemic conditions) underwent full-arch rehabilitation with conventional and pterygoid implants after tooth extraction. Case 3 (58-year-old woman) received a combination of conventional, zygomatic, and pterygoid implants; one implant was not used due to angulation. Case 4 (58-year-old woman) with an edentulous maxilla was treated with zygomatic, pterygoid, and conventional implants. All cases achieved 100% implant survival during follow-up periods ranging from 10 to 13 months. Prosthetic function remained stable in all patients, with no biological or mechanical complications reported. Smooth-collar (tissue-level) implants demonstrated predictable short-term outcomes in the rehabilitation of severely atrophic maxillae, including anatomically challenging regions such as zygomatic and pterygoid areas. Further studies with larger samples and longer follow-up are needed to confirm these findings.

A systematic review was also conducted following the Preferred Reporting Items for Systematic reviews and Meta-Analyses (PRISMA) guidelines to evaluate the performance and safety of dental implants with a smooth/machined collar (tissue-level), with a particular focus on complex maxillary rehabilitations using zygomatic and pterygoid implants. Searches were performed in PubMed, SciELO, BVS, and Google Scholar for articles published between January 1, 2010, and January 28, 2025. Eligible studies included prospective and retrospective clinical trials, case series, and systematic reviews evaluating implants with a machined collar height > 3 mm in edentulous patients. Key outcomes assessed were implant survival rate, marginal bone loss, biofilm control, and incidence of complications.

## Introduction

Modern implantology is founded on the principle of osseointegration and has demonstrated high long-term clinical predictability [1,2]. Despite these favorable outcomes, biological complications - particularly peri-implant mucositis and peri-implantitis - remain significant challenges, especially in complex rehabilitations or in patients with limited access to adequate oral hygiene [3,4].

The interface between the implant and peri-implant soft tissues plays a critical role in controlling microbial colonization and maintaining tissue health. In this context, implant design - particularly at the cervical region - has been increasingly investigated. Implants with a polished or smooth collar have been proposed as a strategy to reduce biofilm accumulation, modulate inflammatory response, and improve soft tissue sealing [5,6].

Experimental and clinical studies suggest that the cervical design of implants directly influences marginal bone preservation. Smooth-collar implants have been associated with reduced buccal bone resorption when compared to rough-neck designs [7,8]. Additionally, long-term clinical data indicate a lower incidence of peri-implantitis and marginal bone loss in implants with machined collars, with follow-up periods extending up to 10 years [9,10].

In patients with atrophic maxillae who are candidates for graftless rehabilitation, zygomatic and pterygoid implants offer an alternative by anchoring in distant anatomical structures. However, these implants often present long transmucosal paths and emerge outside the alveolar ridge, which may increase the risk of biofilm accumulation and soft tissue complications. In such scenarios, the use of smooth-neck implants may provide advantages by reducing bacterial adhesion and enhancing mucosal sealing, thereby minimizing chronic inflammation [11,12].

Therefore, the present study aims to report clinical cases of maxillary rehabilitation in atrophic maxillae using smooth-neck implants in zygomatic and pterygoid configurations. Additionally, a systematic review of the clinical literature was conducted to evaluate the performance and safety of implants with a smooth (machined) collar, with a particular focus on their application in complex maxillary rehabilitations.

## Case presentation

This study consists of a series of clinical case reports describing the rehabilitation of atrophic maxillae using smooth-neck dental implants, including zygomatic and pterygoid implants. Patients were selected based on the presence of severe maxillary atrophy requiring full-arch rehabilitation without bone grafting. Clinical and radiographic evaluations, including cone-beam computed tomography (CBCT), were performed for diagnosis and treatment planning.

All surgical procedures followed standardized protocols, including implant placement under local anesthesia, aiming for high primary stability to allow immediate loading when insertion torque exceeded 45-50 Ncm. Smooth/machined collar implants (tissue-level) were used in all zygomatic and pterygoid cases. Outcome assessment included implant stability, prosthetic function, and the presence of biological or mechanical complications during follow-up periods ranging from 10 to 13 months.

Case 1

**Figure 1:**
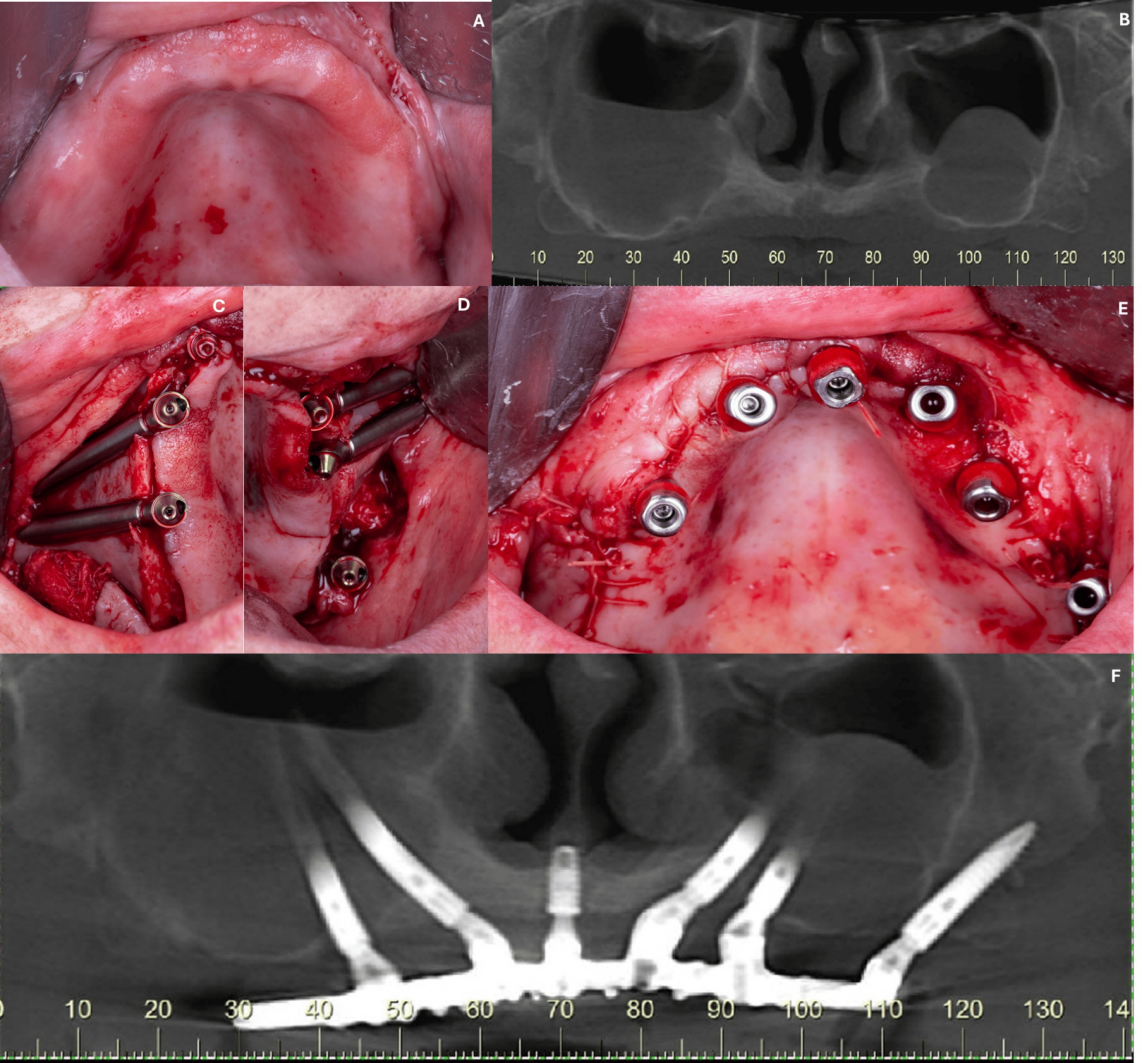
(A) Initial clinical condition; (B) initial radiograph exam showing the atrophic bone condition of the maxilla; (C, D) surgery was performed to place the dental implants; (E) clinical condition after implant placement; (F) radiographic condition of the prosthesis supported by implants after 10 months of follow-up

A 65-year-old female patient, systemically healthy, required full rehabilitation of the atrophic maxilla. Clinical and radiographic evaluation revealed a premaxilla with reduced bone thickness and the posterior region of the maxilla with a pneumatized maxillary sinus (Figures 1A, 1B). For this case, a treatment plan involving the placement of six implants was adopted, as follows: four zygomatic implants placed bilaterally (Zygomatic Plus Ø 4.0 platform diameter, Ø 4.0 thread diameter, 50 mm (ILMZ 4050)/42.5 mm (ILMZ 4042)/50 mm (ILMZ 4050)/52.5 mm (ILMZ 4052) lengths, Cone Morse, S.I.N. Implant System, Brazil), one conventional implant (Cone Morse 11.5°, Strong SW, Ø 4.5 x 8.5 mm, S.I.N. Implant System), and one pterygoid implant (Cone Morse Epikut PTG Plus Ø 4.2 x 22.0 mm, ILMP 4222N, S.I.N. Implant System) (Figures 1C-1E). The zygomatic and pterygoid implants featured a smooth/machined cervical surface (tissue-level). As the implants presented higher primary stability (>50 Ncm), an immediate loading was performed, and the patient has been functionally using the prosthesis for 10 months (Figure 1F). The following abutments were used in this case: angled abutment, Ø 4.8 x 3.0 mm, 45° (MAAM 4843I, S.I.N. Implant System) and straight mini abutment, Ø 4.8 x 3.5 mm (MAMU 4835, S.I.N. Implant System).

Case 2

**Figure 2:**
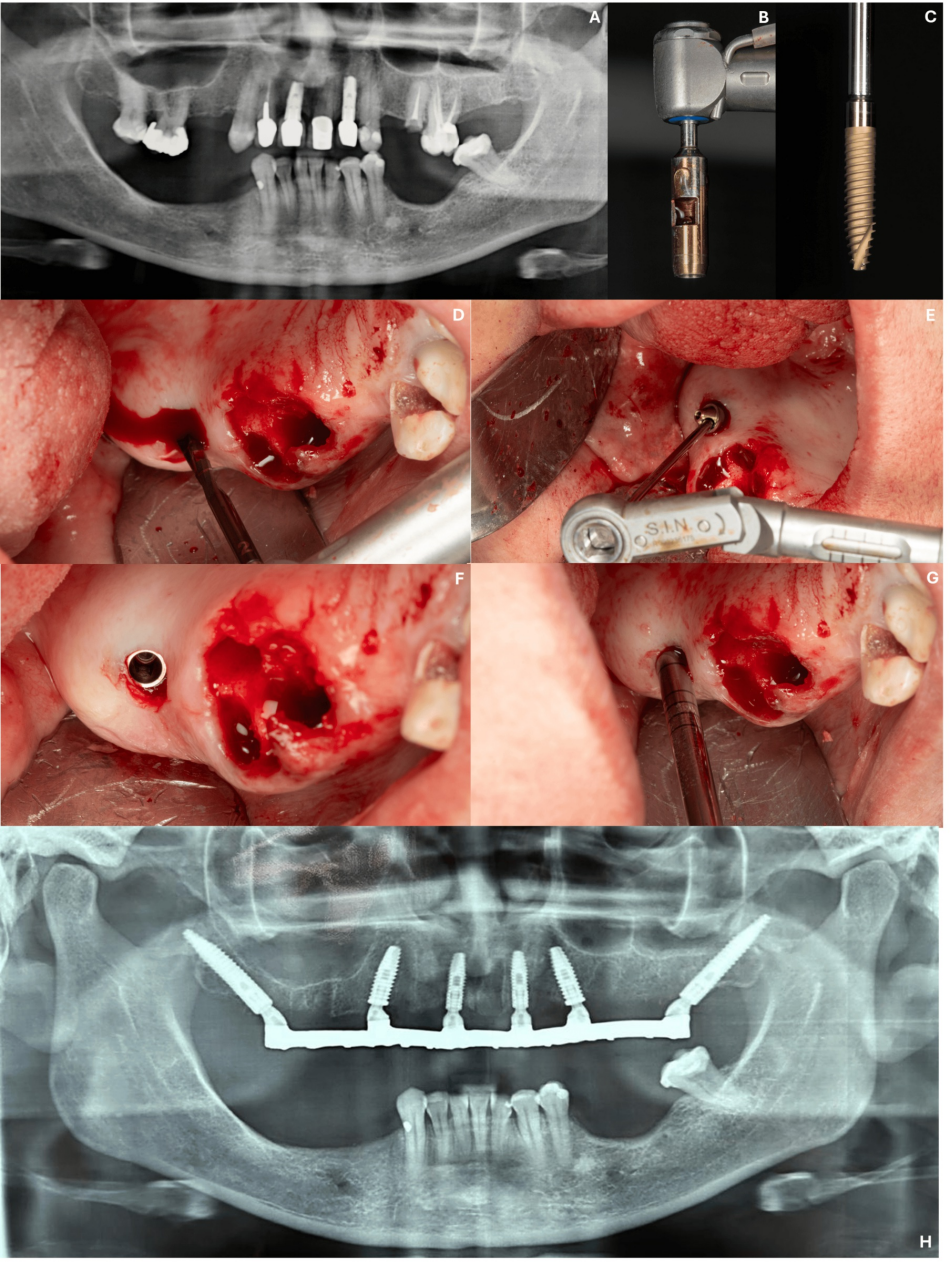
(A) Radiographic analysis showed that the patient presented a lot of teeth with an unfavorable prognosis; (B-G) four conventional implants and two pterygoid implants were placed to support a maxillary full-arch prosthesis; (H) radiographic condition of the prosthesis in function for 13 months without complications

A 58-year-old female patient presented with non-restorable maxillary teeth due to extensive carious lesions, which made the prognosis for a tooth-supported rehabilitation challenging (Figure 2A). Additionally, the patient reported systemic conditions including hypertension, gastritis, and well-controlled diabetes. The treatment plan included the extraction of all maxillary teeth and the preservation of two existing implants. Two conventional implants (Cone Morse Strong SW, Ø 4.5 x 13.0 mm and Ø 3.8 x 13.0 mm) (Figure 2B) and two pterygoid implants (Cone Morse 16° Epikut PTG Plus, Ø 4.5 x 24.0 mm - ILMP 4524EN, and Cone Morse 16° Epikut PTG Plus Ø 4.0 x 24.0 mm - ILMP 4024EN, S.I.N. Implant System) (Figure 2C) were placed to support a maxillary full-arch prosthesis. The following abutments were used in this case: angled mini abutment Ø 4.8 x 3.0 mm, 45° - MAAM 4843l; angled mini abutment Ø 4.8 x 2.0 mm, 45° - MAAM 4842l; and straight mini abutments Ø 4.8 x 2.5 mm - MAMU 4825 (S.I.N. Implant System). Implant placement occurred in fresh extraction sockets in the anterior region and in native bone in the maxillary tuberosity area posteriorly (Figures 2D-2G). The lower arch was rehabilitated by a partial removable prosthesis. The prosthesis has been in function for 13 months without early complications (Figure 2H).

Case 3

**Figure 3:**
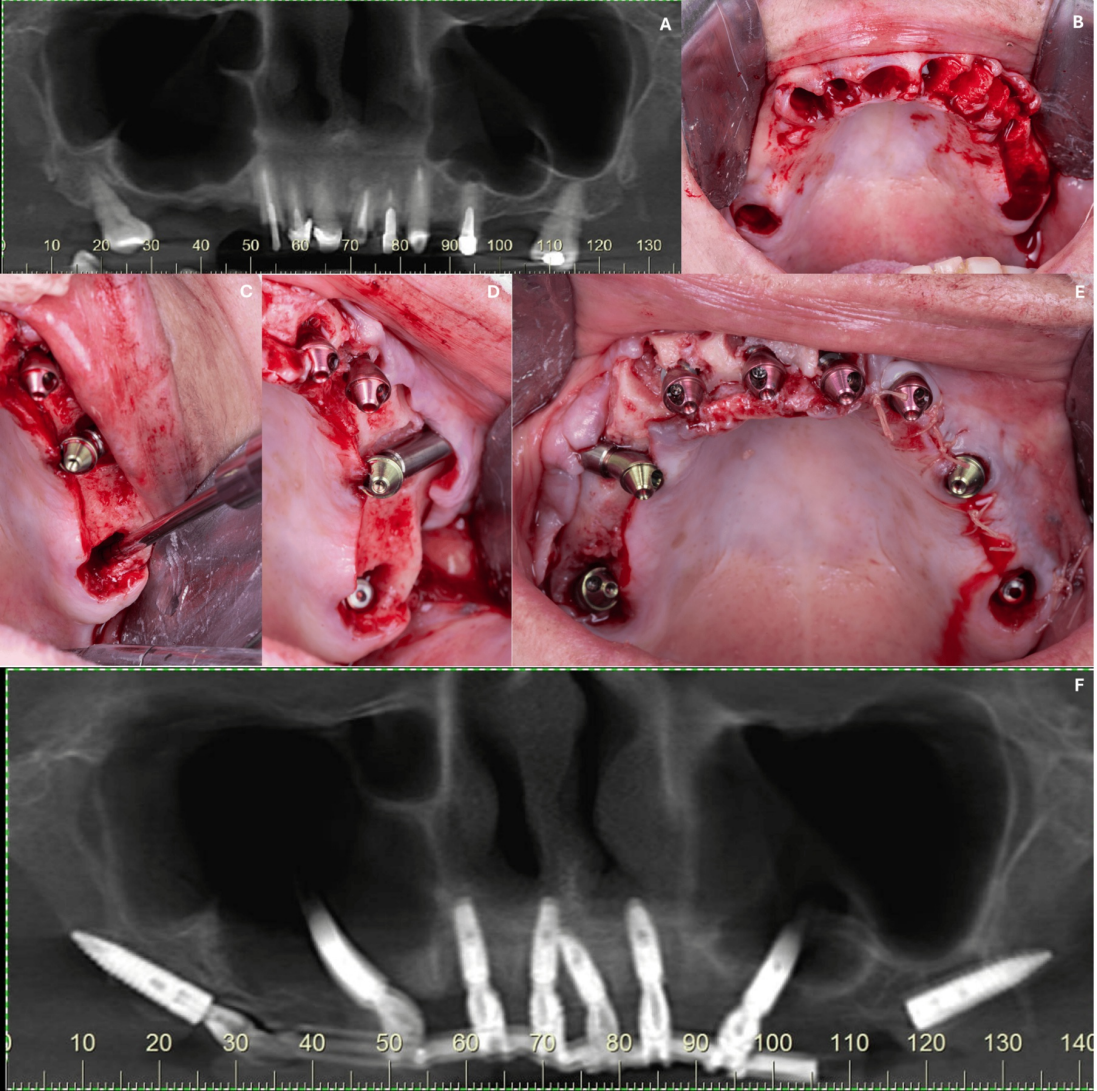
(A) Radiographic imaging showing teeth with inadequate direct and indirect restorations and large maxillary sinuses; (B) because of this, the total tooth extraction was indicated; (C-E) a full-arch maxillary rehabilitation supported by eight implants was proposed, including four conventional implants placed in the anterior region in fresh extraction sockets, two zygomatic implants, and two pterygoid implants; (F) radiographic condition of the prothesis supported by dental implants after 13 months of the implant loading

This case report refers to a 58-year-old female patient who was hypertensive and a light smoker (less than 10 cigarettes per day). The patient complained of esthetic issues related to her maxillary teeth. Clinical and radiographic examinations revealed teeth with inadequate direct and indirect restorations due to an extended period of prior treatments (Figure 3A). Maxillary atrophy was observed, particularly due to the patient’s large maxillary sinuses, as well as the need for extraction of all remaining maxillary teeth (Figures 3A, 3B). A full-arch maxillary rehabilitation supported by eight implants was proposed, including four conventional implants placed in the anterior region in fresh extraction sockets (Cone Morse 11.5° Strong SW, Ø 3.8 x 11.0 mm, S.I.N. Implant System), two zygomatic implants (Zygomatic Plus, Ø 4.0 x 45.0 mm - ILMZ 4045 and Ø 4.0 x 42.5 mm ILMZ 4042N, S.I.N. Implant System), and two pterygoid implants (Cone Morse 16° Epikut PTG Plus, Ø 4.5 x 20.0 mm, ILMP 4520EN, S.I.N. Implant System) placed in healed ridges with native bone (Figures 3C-3E). A full-arch prosthesis was installed and supported by seven implants; the eighth implant (right-side pterygoid) was not used due to excessive angulation after surgery (Figure 3F). The following abutments were used in this case: angled mini abutment Ø 4.8 x 2.5 mm, 30° (MAMA 3025I); angled mini abutment Ø 4.8 x 2.5 mm, 17° (MAMA 1725I); straight mini abutments Ø 4.8 x 2.5 mm (MAMU 4825); angled mini abutment Ø 4.8 x 3.0 mm, 30° (MAAM 4833I); angled mini abutment Ø 4.8 x 2.0 mm, 30° (MAAM 4832I); and angled mini abutment Ø 4.8 x 3.0 mm, 45° (MAAM 4843I). The prosthesis has been functioning for 13 months without complications or adverse events (Figure 3F).

Case 4

**Figure 4:**
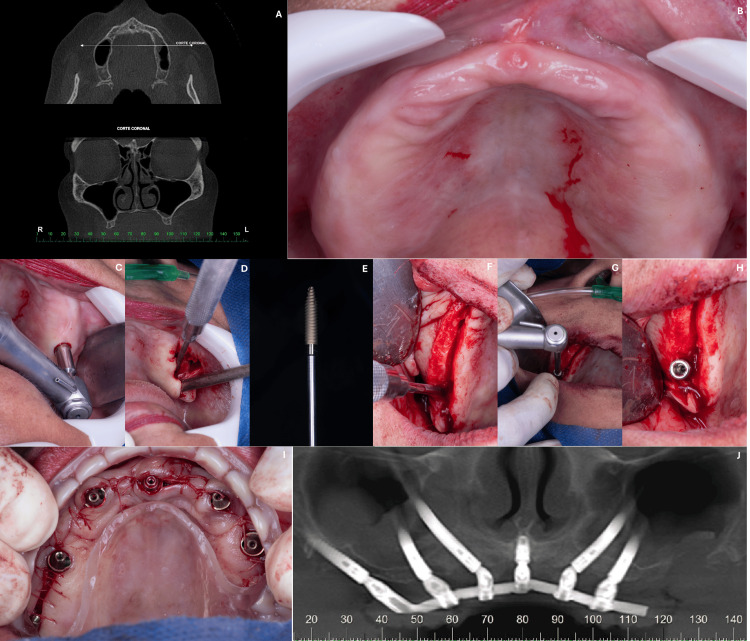
(A, B) Clinical and radiographic assessments revealed a severely atrophic maxilla associated with enlarged maxillary sinuses; (C-H) the treatment plan included the placement of six implants to support a maxillary full-arch prosthesis: two pterygoid implants, three zygomatic implants placed posteriorly, and one conventional implant; (J) the prosthesis was installed and supported by all implants and has been in function for 13 months without complications

A 58-year-old female patient, with a history of hypertension, presented with a completely edentulous maxilla and dissatisfaction with her current upper dentures. Clinical and radiographic assessments revealed a severely atrophic maxilla associated with enlarged maxillary sinuses (Figures 4A, 4B). The treatment plan included the placement of six implants to support a maxillary full-arch prosthesis: two pterygoid implants (Cone Morse 16° Epikut PTG Plus - ILMP 4047N and Cone Morse 16° Epikut PTG Plus Ø 4.2 x 24.0 mm - ILMP 4224EN), three zygomatic implants placed posteriorly (Zygomatic Plus, Ø 4.0 x 55.0 mm - ILMZ 4055N, two Ø 4.0 x 50.0 mm - ILMZ 4050N), and one conventional implant (Cone Morse 11.5° Strong SW, Ø 3.8 x 11.0 mm, S.I.N. Implant System) placed in the anterior region (Figures 4C-4I). The following abutments were used in this case: angled mini abutment Ø 4.8 x 2.0 mm, 45° - MAAM 4842l; and angled mini abutment Ø 4.8 x 3.0 mm, 45° (MAAM 4843I). The prosthesis was installed and supported by all implants and has been in function for 13 months without complications (Figure 4H).

## Discussion

Literature review

Study Description

In addition to the clinical data obtained through a case series, we have gathered relevant clinical literature data through systematic searches in major databases to provide clinical and radiographic evidence of the performance and safety of smooth/machined surface (tissue-level) implants. Eligible articles published in peer-reviewed journals from January 1, 2010, to January 28, 2025, were considered. The selected body of evidence encompasses critical evaluation parameters (survival rates, osseointegration failures, adverse effects, among others) that support the implant system’s claim regarding device design differences and the risks created by these design differences.

Search Strategy and Study Selection

Systematic reviews are the standard method for synthesizing evidence in healthcare because of their methodological rigor. They are used to support the development of clinical practice guidelines and inform clinical decision-making [13].

**Figure 5:**
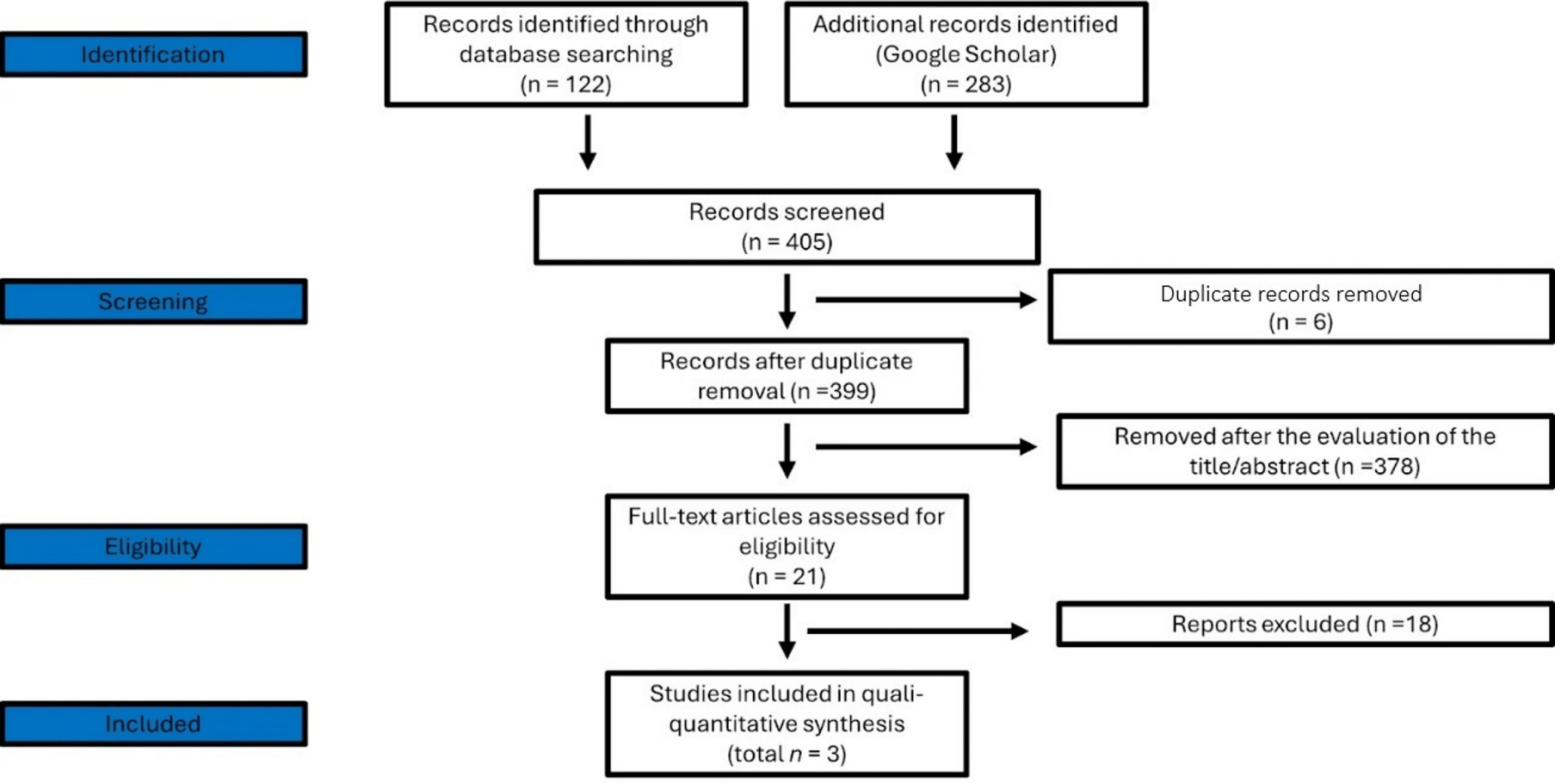
Flowchart of the study selection

Here, the systematic review was conducted according to the Preferred Reporting Items for Systematic Reviews and Meta-Analyses (PRISMA) guidelines (www.prisma-statement.org), as shown in Figure 5. Independent searches were conducted in the following databases: Medline via PubMed, SciELO, BVS, and Google Scholar, searching for articles published between January 1, 2010, and January 28, 2025. The search strategy included combinations of keywords, following the syntax rules of each database, as shown in Table 1.

**Table 1:** Search strategy and bibliographic databases used to retrieve the articles falling into the scope of this systematic review

Bibliographic databases (primary sources)	Search strategy (descriptors and Boolean operators)	Records retrieved	Articles included in the qualitative synthesis
BVS (Virtual Health Library) filter(s): 2010-2025; LILACS; BBO; Western Pacific full-text articles	(smooth collar implant) or (machined surface implant) and (dental implant) and (implant survival) https://pesquisa.bvsalud.org/portal/?u_filter%5B%5D=fulltext&u_filter%5B%5D=db&u_filter%5B%5D=mj_cluster&u_filter%5B%5D=type_of_study&u_filter%5B%5D=la&fb=&output=site&lang=en&from=1&sort=&format=summary&count=20&page=1&tab=2&range_year_start=2010&range_year_end=2025&skfp=&index=&q=%28smooth+collar+implant%29+or+%28machined+surface+implant%29+and+%28dental+implant%29+and+%28implant+survival%29&where=&filter%5Bfulltext%5D%5B%5D=1&filter%5Bdb%5D%5B%5D=WPRIM&filter%5Bdb%5D%5B%5D=LILACS&filter%5Bdb%5D%5B%5D=BBO&range_year_start=2010&range_year_end=2025&filter%5Bfulltext%5D%5B%5D=1&filter%5Bfulltext%5D%5B%5D=1&filter%5Bfulltext%5D%5B%5D=1	9	0
Medline via PubMed filter(s): 2010-2025; full-text articles	(smooth collar implant) or (machined surface implant) and (implant survival) https://pubmed.ncbi.nlm.nih.gov/?term=%28smooth+collar+implant%29+or+%28machined+surface+implant%29+and+%28implant+survival%29&filter=years.2010-2025	112	3
SciELO (Scientific Electronic Library Online) filter(s): none	(smooth collar implant) OR (machined collar implant) https://search.scielo.org/?q=%28%28smooth+collar+implant%29%29+OR+%28machined+collar+implant%29&lang=en&count=15&from=0&output=site&sort=&format=summary&fb=&page=1&q=%28smooth+collar+implant%29+OR+%28machined+collar+implant%29&lang=en&page=1	1	0
Google Scholar filter(s): since 2024	Additional searches for articles published in journals not indexed in major databases [(smooth collar implant) or (machined surface implant) and (implant survival) and (implant performance)] *As the number of retrieved articles published in non-indexed journals was too high to assess, in this database we selected only those published since 2024 (pterygoid implants OR pterygomaxillary implants) AND (atrophic maxilla) https://scholar.google.com.br/scholar?as_ylo=2024&q=(pterygoid+implants+OR+pterygomaxillary+implants)+AND+(atrophic+maxilla)&hl=en&as_sdt=0,5	283	0

Formulating relevant and precise questions that can be answered in a systematic review can be a complex task. A structured approach for framing questions that uses five components may help facilitate the process. This approach is commonly known by the acronym “PICOS,” where each letter refers to a component: (P) the patient population or the disease being addressed, (I) the interventions or exposure, (C) the comparator group, (O) the outcome or endpoint, and (S) the study design.

The initial screening excluded articles with titles and abstracts that were not related to the scope of the study and that met at least one of the exclusion criteria. The next step was a detailed analysis of the selected articles to examine which met all the inclusion criteria. When the information in the title or abstract was insufficient to make a decision, the full article was read in full for eligibility. Articles without an abstract were read in full for analysis of eligibility (Figure 5).

Eligibility Criteria for Selection

The articles were selected based on the title and abstract, considering the following inclusion criteria: edentulous patients (P: participant); tissue-level implant rehabilitation, particularly smooth/machined collar implants with a collar height > 3.0 mm (I: intervention); no comparison specified (C: not applicable); osseointegration (O: outcome); and prospective or retrospective clinical studies/clinical radiographic studies/systematic reviews/case series (S: study design). In addition to the literature in English, we also considered publications in any other language. If potentially relevant studies in other languages were found, they were also considered eligible for full-text analysis. The exclusion criteria were as follows: articles out of the scope of this systematic review, in vitro studies with a very low level of evidence, articles with poor methodological quality, and articles missing relevant information to the purpose of the study.

Databases Screened for Eligible Studies

This clinical evaluation is based on the currently available scientific literature. A systematic review was performed in compliance with rigorous methodological criteria for the selection of high-quality articles.

For the development of this report, the search was carried out in major scientific research databases: Medline, PubMed, Central, LILACS, and SciELO. The keywords used contemplate the purpose of this report, and the Boolean combinations allowed favorable and unfavorable papers to be unbiasedly identified without any preference between them.

The materials and methods of each study, sample size, products, and brands were carefully examined. The selection of articles took place in several stages. The first stage consisted of the search for articles indexed in relevant databases, such as PubMed, SciELO, and BVS (VHL). The literature searches in these databases were performed using keywords and combinations of "Boolean" operators based on the scope of the systematic review. The second stage consisted of screening all titles and abstracts for eligibility based on inclusion and exclusion criteria. In the third stage, the studies were analyzed in full for the performance and safety of the tested dental implants.

The PubMed database includes over 26 million citations for biomedical literature from Medline, life sciences journals, and online books. In this database, citations can contain links to the full content on PubMed Central or to publishers' websites. PubMed was developed by the National Center for Biotechnology Information (NCBI) and is maintained by the National Library of Medicine of the United States of America. Generally, citations, abstracts, and full-text articles indexed in PubMed fall within the following related areas: biomedical and health, natural sciences, behavioral sciences, chemistry, and bioengineering. PubMed provides access to relevant sites and links for molecular biology sources of NCBI (https://www.ncbi.nlm.nih.gov/pubmed/).

Quality Assessment of Selected Studies

To assess the quality of evidence, strength of the recommendations of the selected studies, and quality of the study design, the Strength of Recommendation Taxonomy (SORT) framework was used [14]. A summary of the quality of evidence, strength of scientific recommendation, and quality of the studies included [15-17] in the systematic review is presented in Table 2.

**Table 2:** Quality of evidence, strength of scientific recommendation, and quality of the studies included in the systematic review, according to the Strength of Recommendation Taxonomy (SORT) framework

Article number	Article author	Strength of recommendation	Quality of the evidence	Type of study
1	Menchini-Fabris et al., 2025 [15]	B	Level 2	Level 2
2	Pande and Bhoge, 2024 [16]	A	Level 1	Level 1
3	Signorini et al., 2021 [17]	A	Level 1	Level 1

Results and discussion

Case Series

The clinical cases presented demonstrate the feasibility and success of using implants with machined (smooth) collars in full-arch rehabilitation of the atrophic maxilla, even in patients with diverse systemic conditions and challenging anatomical situations. The use of zygomatic, pterygoid, and conventional implants - all featuring smooth collars - enabled a safe and effective approach for immediate or early prosthetic loading, with clinical follow-ups ranging from 10 to 13 months without significant complications.

The choice of smooth collars was crucial to minimize bacterial biofilm accumulation, especially in anatomical regions where hygiene maintenance is more difficult, such as the posterior maxilla with thicker mucosa, as discussed in the literature [17-19]. The smooth cervical surface promoted better soft tissue adaptation, facilitating peri-implant health and preventing peri-implantitis, consistent with the high success rates observed in these cases.

Furthermore, the clinical reports showed that combining implants with different designs and dimensions, individually tailored to each patient’s anatomy and bone condition, allowed for balanced force distribution and prosthesis stability. This aligns with evidence emphasizing the importance of implant design and the soft tissue interface for long-term treatment success [16,17,20].

Another important aspect was the possibility of immediate or early loading due to the high primary stability observed in the cases (>40 Ncm of insertion torque), which facilitated functional and esthetic rehabilitation in a short period, with no reported complications even in patients with comorbidities such as hypertension and well-controlled diabetes. However, in one case, one implant was not rehabilitated due to the higher angulation. The planning of the implant placement with CBCT and the use of guided surgery may improve the implant placement angulation. This reinforces the effectiveness of the technique and components used, consistent with studies reporting positive outcomes using implants with machined surfaces in complex cases [21,22].

Although the follow-up period is relatively short, the initial results are promising and suggest that the use of machined collars in zygomatic, pterygoid, and conventional implants represents a safe and reliable clinical strategy for the rehabilitation of the atrophic maxilla, with potential for long-term peri-implant health maintenance. Future studies with longer follow-up are essential to confirm these findings and establish optimized protocols for different patient profiles.

Qualitative Synthesis of Selected Studies

A rigorous search strategy yielded 405 records from major databases and Google Scholar. After screening titles and abstracts, 21 articles were assessed in full text, of which three met the inclusion criteria for qualitative synthesis. Excluded studies primarily consisted of in vitro research, articles outside the scope, or those lacking relevant clinical data (Table 3).

**Table 3:** Eligible studies selected in the systematic review addressing the performance and safety of smooth-collar implants in the rehabilitation of totally or partially edentulous patients (January 2010 to January 2025)

Year and author	Study design	Sample features	Study implants	Predicate (brand)	Diameter	Length	Collar configuration	Follow-up	Performance	Safety	Adverse effects
2025, Menchini-Fabris et al. [15]	Retrospective radiologic and clinical study (3 to 5 years)	48 patients composed the total sample - no information was reported specifically for the patient sample of pterygoid implants	6 pterygoid implants	JDentalCare (JD Pterygo, Modena, Italy)	3.3 to 4.0 mm	13 to 18 mm	Machined 3.5 mm collar height	3 to 5 years	None of the pterygoid implants (5 in the bilateral group and 1 in the unilateral group) failed, with a 100% success rate of the JD Pterygo implant	No intra- and postoperative complications directly associated with these implants were reported. Some implant complications were observed for the other group of implants, such as mechanical failure of the prosthesis due to implant failure, peri-implant mucositis, and acute rhinosinusitis	No adverse effects were reported for these implants
2024, Pande and Bhoge [16]	Systematic review; 5 selected studies (2013-2022)	1,279 patients, with ages ranging from 38 to 77 years	Not reported	Nobel-Biocare/Bioline Dental Implants/JDentalCare	4 mm	7 to 22 mm	Machined 3.5 mm collar height for JDentalCare implants	11 months to 10 years	The survival rate in the included studies ranged from 88.06% to 100%. The survival rate of the JD Pterygo implants (3.5 mm machined collar) was 100%	Postoperative complications like mucositis, fractured prosthesis, chipping of ceramic, peri-implant mucositis, and mobility, bleeding, or discomfort were observed in 1.02% (13 of 1,279) of the patients	No adverse effects other than the reported complications
2021, Signorini et al. [17]	Prospective cohort study	15 patients with severe atrophy of the posterior maxilla, with a mean age of 61 years (51-77 years)	15 pterygoid implants	JDentalCare (JD Pterygo)	4.0 mm	13 to 20 mm	Machined 3.5 mm collar height	1 year	All implants were well-positioned in the bone site, with no implant failure, pain, or peri-implant radiolucency. Survival and success rates within the follow-up period were 100%	During the follow-up, no major complications were found for the dental protheses or the implants. All prostheses were stable with no implant loss	No adverse effects were reported for these implants

The selected studies comprised one systematic review, one prospective cohort, and one retrospective clinical radiographic study. Collectively, they evaluated 21 smooth-collar implants manufactured by JDentalCare (JD Pterygo, Modena, Italy), with patient ages ranging from 38 to 77 years.

Rationale for the Use of Machined (Smooth) Collar Implants

The implant collar, or crest module, functionally differs from the implant body by focusing on minimizing bacterial invasion rather than force transmission [19]. Machined (smooth) collars were specifically designed to reduce plaque accumulation and facilitate hygiene maintenance, particularly beneficial in challenging anatomical regions such as the posterior maxilla, where mucosal thickness is greater [18]. These smooth surfaces in contact with soft tissue have demonstrated excellent biocompatibility and a high cumulative success rate - up to 97.9% over 10 years [15]. The topographic and chemical properties of implant surfaces also play a key role in tissue integration [20,21].

Survival Rates and Radiographic Outcomes

The pooled data from the included studies indicate a 100% survival rate of tissue-level smooth-collar implants over follow-up periods ranging from one to five years [16,22]. Menchini-Fabris et al. [15] reported no failures or implant-related complications in a retrospective study of six pterygoid implants with 3.5 mm smooth collars, followed for up to five years. Similarly, Signorini et al. [17] documented no implant loss and high prosthesis stability in a cohort of 15 patients rehabilitated with JDentalCare smooth-collar implants over one year. These findings support the long-term reliability of smooth-collar implants in atrophic maxillary rehabilitation.

Enhanced Biofilm Control With Smooth Collars

Supporting these clinical outcomes, Otsuki et al. [22] demonstrated in an ex vivo model that biofilms on machined surface implants are more effectively removed by conventional decontamination methods than biofilms on rough surfaces. This reduced biofilm retention likely contributes to lower rates of peri-implant inflammation and infection in clinical practice. However, such implants are contraindicated in patients with poor oral hygiene, as per device instructions for use (IFU) guidelines.

Effects on Marginal Bone Loss and Other Clinical Parameters

Evidence suggests that implant collar design significantly influences marginal bone preservation and peri-implant health. Sánchez-Siles et al. [23] observed significantly lower marginal bone loss around smooth-neck implants (1.18 ± 1.39 mm) compared to rough-neck implants (2.41 ± 1.35 mm) after 10 years (p < 0.001). Additionally, peri-implantitis incidence was markedly reduced in the smooth-neck group (2.92% vs. 14.41%, p < 0.001). These findings align with previous studies [17,22-24], which reported better marginal bone stability and lower probing depths with machined collars compared to microtextured or microthreaded collars. While the smooth-collar design may slightly compromise esthetics [24], the clinical benefits of improved tissue health and patient-reported quality of life outweigh this drawback in many rehabilitations, particularly in atrophic maxillae.

Implant Dimensions and Relevance to the Subject Device

The implants studied featured diameters of approximately 4.0 mm and lengths ranging from 13 to 20 mm, consistent with the specifications of the Epikut PTG Plus implant system evaluated in the present case series.

Safety and Adverse Events

Minor complications such as prosthesis mechanical failure and peri-implant mucositis were reported but were associated with other implant groups. Pande and Bhoge [16] reported low complication rates (~1%) in a large systematic review involving 1,279 patients, reinforcing the established safety profile of these implants when used appropriately and with proper clinical expertise.

Limitations

A limitation of this study is the relatively short follow-up period (10-13 months), which does not allow definitive conclusions regarding long-term prognosis, implant survival, and biological complications such as peri-implantitis. Future studies with extended follow-up are necessary to validate these preliminary findings.

## Conclusions

Within the limitations of this study, including the short follow-up period and limited number of included studies, smooth-collar implants demonstrated high short-term survival rates and low complication rates in the rehabilitation of atrophic maxillae. The findings suggest that this implant design may offer biological advantages in complex anatomical conditions, particularly in reducing biofilm accumulation and supporting peri-implant tissue stability. However, the current level of evidence remains limited, and well-designed long-term clinical studies are required to confirm these outcomes and establish definitive clinical protocols.
